# The Barley *S-Adenosylmethionine Synthetase 3* Gene *HvSAMS3* Positively Regulates the Tolerance to Combined Drought and Salinity Stress in Tibetan Wild Barley

**DOI:** 10.3390/cells9061530

**Published:** 2020-06-23

**Authors:** Imrul Mosaddek Ahmed, Umme Aktari Nadira, Cheng-Wei Qiu, Fangbin Cao, Zhong-Hua Chen, Eva Vincze, Feibo Wu

**Affiliations:** 1 Department of Agronomy and Zhejiang Key Laboratory of Crop Germplasm, College of Agriculture and Biotechnology, Zijingang Campus, Zhejiang University, Hangzhou 310058, China; imrulbau@gmail.com (I.M.A.); kbdnadira@yahoo.com (U.A.N.); 3130100260@zju.edu.cn (C.-W.Q.); caofangbin@zju.edu.cn (F.C.); 2 Plant Physiology Division, Bangladesh Agricultural Research Institute, Gazipur 1701, Bangladesh; 3 Jiangsu Co-Innovation Center for Modern Production Technology of Grain Crops, Yangzhou University, Yangzhou 225009, China; 4 School of Science and Health, Hawkesbury Institute for the Environment, Western Sydney University, Penrith, NSW 2751, Australia; z.chen@uws.edu.au; 5 Department of Molecular Biology and Genetics, Aarhus University, Fosøgsvej 1, DK-4200 Slagelse, Denmark; eva.vincze@mbg.au.dk

**Keywords:** Tibetan wild barley, barley stripe mosaic virus-based virus-induced gene silencing (BSMV-VIGS), combination of drought and salinity, polyamine, proteomics

## Abstract

Drought and salinity are two of the most frequently co-occurring abiotic stresses. Despite recent advances in the elucidation of the effects of these stresses individually during the vegetative stage of plants, significant gaps exist in our understanding of the combined effects of these two frequently co-occurring stresses. Here, Tibetan wild barley XZ5 (drought tolerant), XZ16 (salt tolerant), and cultivated barley *cv*. CM72 (salt tolerant) were subjected to drought (D), salinity (S), or a combination of both treatments (D+S). Protein synthesis is one of the primary activities of the green part of the plant. Therefore, leaf tissue is an important parameter to evaluate drought and salinity stress conditions. Sixty differentially expressed proteins were identified by mass spectrometry (MALDI-TOF/TOF) and classified into 9 biological processes based on Gene Ontology annotation. Among them, 21 proteins were found to be expressed under drought or salinity alone; however, under D+S, 7 proteins, including S-adenosylmethionine synthetase 3 (SAMS3), were exclusively upregulated in drought-tolerant XZ5 but not in CM72. *HvSAMS3* carries both N-terminal and central domains compared with *Arabidopsis* and activates the expression of several ethylene (ET)-responsive transcription factors. *HvSAMS3* is mainly expressed in the roots and stems, and HvSAMS3 is a secretory protein located in the cell membrane and cytoplasm. Barley stripe mosaic virus-based virus-induced gene silencing (BSMV-VIGS) of *HvSAMS3* in XZ5 severely compromised its tolerance to D+S and significantly reduced plant growth and K^+^ uptake. The reduced tolerance to the combined stress was associated with the inhibition of polyamines such as spermidine and spermine, polyamine oxidase, ethylene, biotin, and antioxidant enzyme activities. Furthermore, the exogenous application of ethylene and biotin improved the tolerance to D+S in BSMV-VIGS:HvSAMS3-inoculated plants. Our findings highlight the significance of *HvSAMS3* in the tolerance to D+S in XZ5.

## 1. Introduction

Drought and salinity are major abiotic stressors in plant crops that limit the overall growth and yield worldwide. The resistance and adaptation of plant crops to drought and salt stress are involved in the perception of stress signals and the expression of stress-responsive genes. Thus, breeding scientists exert much effort to improve crop tolerance to abiotic stresses considering climate change, the scarcity of fresh water, and environmental pollution [1]. The most effective approach to fight against drought/salinity is through the development of tolerant crop varieties [2].

Recently, high-throughput crop proteomics has become the method of choice to conduct large-scale, quantitative, and reproducible proteomic profiling of the abiotic stress response. The utilization of proteomics to investigate plant stress has been an increasingly important method to identify possible candidate genes that can be used for the genetic enhancement of plants against stresses [3]. Several recent studies have shown the changes in the proteome profiles in response to salinity and drought stresses [4,5,6]. Proteins induced by drought or salinity that are involved in photosynthesis, signaling pathways, and oxidative stress detoxification have been identified [7,8]. For example, salinity treatment of *Arabidopsis thaliana* seedlings induced phosphorylation, which affects the expression of aquaporins and mitrogen-activated protein kinase kinases (MAPKK), among others [9]. Under water-stressed conditions, plants survive by chaperone synthesis and using the oxidative stress tolerance mechanism [10]. Ashoub et al. [11] also examined drought- or salt-tolerant and -susceptible barley varieties under abiotic stresses and conducted proteomic/metabolomic analyses.

S-adenosylmethionine synthetases (SAMSs) play roles in plant development and the stress response [12]. SAMSs play central roles in many cellular biochemistry reactions and are involved in the biosynthetic pathways of ethylene, polyamines (PAs) [13], and biotin [14], interacting with physiological processes and stress responses [15,16]. Ethylene biosynthesis is performed via the activities of 1-aminocyclopropane-1-carboxylate (ACC) synthase and ACO (ACC oxidase) in plants, spermidine, and spermine biosynthesis through the activity of the SAM decarboxylase, and biotin biosynthesis through the activity of 7,8-diaminope-largonic acid aminotransferase. Ethylene is regarded as a stress-responsive hormone besides its roles in the regulation of plant growth and development and was able to improve K^+^ uptake in plant [13]. In drought and salt-tolerant plants, ethylene production was lower, which was found still higher in the most sensitive one. Moreover, they did not observe any competition between polyamines and ethylene biosynthesis for their common precursor, S-adenosylmethionine synthetase (SAMS) [14]. The metabolic reactions of ethylene and biotin are considered important for various physiological, morphological, metabolic, and gene expression changes with the development and growth of the plant [14]. Additionally, ethylene and biotin improve plant tolerance to drought and salinity, largely through enhancing the expression of reactive oxygen species (ROS) scavengers by affecting metabolic pathways and maintaining S-adenosylmethionine synthetase (SAMS) in the polyamines biosynthetic pathway is also a precursor for ethylene synthesis [17,18]. Importantly, putrescine (Put), spermidine (Spd), and spermine (Spm), the major PAs in plants, are involved in the regulation of diverse physiological processes, such as plant growth, flower development, senescence, and fruit maturation and development, and they are also involved in responses to abiotic stresses [19]. SAMS is a pervasive enzyme found in different plant tissues, such as vascular tissues and sclerenchyma in Arabidopsis [20], elongated petunia stems [21], rice leaves [22], and the developing ovary in the pea [23]. Additionally, extensive studies have confirmed the functions of SAMS in abiotic stress tolerance in *Nicotiana tobacum* and *Medicago sativa* [24,25]. Furthermore, high levels of SAM and SAMS activity were found to be correlated with lignification during plant defense responses against pathogen and aluminum stresses in *Andropogon virginicus* [26,27]. In rice, the transcript of a single SAMS gene was reported to be accumulated under water stress [28,29]. Importantly, SAMS genes show multiple biological functions. Therefore, they can be considered a target to improve abiotic stress tolerance in plants. However, to date, no information is available on the potential impact of SAMSs in developing tolerance to combined drought and salinity stress.

Wild barley germplasm contains useful genes and serves as a source of new genetic variation for crop improvement [30]. Identifying the genetic resources with high tolerance, as well as uncovering the underlying adaptation mechanisms to combined stresses of salinity and drought at the proteomic level, may provide an essential foundation for future breeding and genetic engineering efforts [7]. Crop plants are especially sensitive to drought stress during the early reproductive stage [31]. However, the capacity to remobilize vegetative reserves seems to be responsible for maintaining the grain growth rate under drought stress [32]. Middle to late drought stress advances leaf senescence, shortened the grain-filling period, and decreases the grain yield and individual grain weight of barley [33]. We previously compared the morphogenetic and physiological effects of combined salinity and drought stresses on the wild and cultivated barley genotypes at the vegetative stage [34]. A question arises regarding whether the Tibetan wild barley genotypes XZ5 and XZ16 are tolerant to the combined stresses of drought and salinity during the vegetative stage at the proteomic level. If so, the proteomic molecular mechanisms of drought and salinity tolerance in barley at the vegetative stage must be identified. However, the proteomic response and molecular level concerning the combined salinity and drought stresses in barley plants remains largely unexplored.

We hypothesized that the expression of *HvSAMS3* is vital to maintain plant drought and salinity stress tolerance in barley. Therefore, in this study, we examined stress-specific proteins in response to the combination of drought and salinity (D+S) in wild barley by comparing the proteomic responses of the two Tibetan wild barley genotypes XZ16 and XZ5 and salinity-tolerant *cv.* CM72 using two-dimensional gel electrophoresis (2-D) and mass spectrometry (MS). We identified and cloned HvSAMS3, a protein being exclusively upregulated in drought-tolerant XZ5 but not in XZ5-derived CM72 under D+S, and virus-induced gene silencing (VIGS) technology was applied to verify the molecular function of this protein in drought and salinity stress tolerance.

## 2. Materials and Methods

### 2.1. Plant Materials and Experimental Design

A greenhouse pot experiment was carried out in Zhejiang University in Hangzhou, China using two Tibetan wild barleys (XZ5, drought-tolerant; XZ16, salinity-tolerant) and one cultivated barley (salinity-tolerant *Hordeum vulgare cv.* CM72) using treatment methods as described in our previous study [35]. Each pot was fertilized with 1 L of basal nutrient solution (BNS). The composition of the BNS was described previously by Wu et al. [36]. The experimental trial was performed using a split-plot design with treatments as the main plot and genotypes as the subplot with six replicates. The soil moisture content was measured daily using an HH2 Moisture Meter (Delta-T Devices, Cambridge, UK). For the ultrastructure leaf and root samples and proteomic studies, the leaf samples were collected at 4% soil moisture content (SMC). The leaves from 20 plants were pooled for each treatment and each replicate for 2-D protein analysis with 3 replicates. All the reagents were of analytical grade (Sigma-Aldrich, St. Louis, MO, USA), and all stock solutions were prepared with deionized water using a Milli-Q system (Millipore, Billerica, MA, USA).

### 2.2. Investigation of Leaf and Root Ultrastructure

Examination of the leaf and root ultrastructure was carried out using a transmission electron microscope (JEOL TEM-1230EX, Japan) according to Chen et al. [16].

### 2.3. Protein Extraction

According to Nadira et al. [37], total protein was extracted by the phenol extraction method. The leaf samples were ground to a fine powder form in liquid nitrogen using a mortar and pestle. The powder was weighted (1 g) and suspended in 5 mL of ice-cold extraction buffer [1.5% (w/v) polyvinylpyrrolidone (PVP), 0.7 M sucrose, 0.1 M KCl, 0.5 M Tris-HCl, pH 7.5, 250 mM ethylene diamine tetraacetic acid (EDTA), complete protease inhibitor cocktail (Roche, Switzerland), 2% (v/v) β-mercaptoethanol, and 0.5% (*w*/*v*) [(3-cholamidopropyl) dimethylammonium]-1-propane sulfonate (CHAPS)] and homogenized at 4 °C for 20 min. Next, 10 mL of Tris-HCl pH 7.5 saturated phenol was added, and the resulting mixture was rehomogenized for 20 min at 4 °C. After centrifugation at 10,000 *g* for 20 min at 4 °C, the upper phenol phase was removed. The lower phase was re-extracted using the same volume of phenol as above and vortexed for 30 s. Proteins in the collected phenol phase were precipitated by the addition of five volumes of 100 mM of ammonium acetate in methanol and were incubated at −20 °C for 3 h. The extract was centrifuged at 10,000 *g* for 20 min at 4 °C, the supernatant was discarded, and the protein pellet was rinsed twice with 0.2% (*w*/*v*) dithiothreitol (DTT) in acetone. At the interval between the two washing steps, the samples were frozen at −20 °C for 60 min. After air drying, the pellet was resuspended in lysis buffer [7 M urea; 2 M thiocarbamide; 4% (*w*/*v*) CHAPS; 20 mM Tris-HCl, pH 7.4, 0.8% (*v*/*v*) Immobilized pH Gradient (IPG) buffer (Amersham Biosciences), 1% (*w*/*v*) dithiothreitol (DTT)], and the mixture was vortexed for 1 h at room temperature. The suspension was centrifuged at 20,000 *g* for 20 min at 4 °C, and the supernatant was collected as protein extract. The protein concentration in the extract was estimated using the BioRad protein assay kit (BioRad, Hercules, CA, USA) and bovine serum albumin (BSA) as the standard following the manufacturer’s instructions.

### 2.4. Two-Dimensional Gel Electrophoresis (2-DE) and Image Analysis

Extracted proteins were separated using two-dimensional gel electrophoresis (2-DE) with isoelectric focusing (IEF) as the first dimension [37]. Protein visualization, image analysis, and quantification were carried out as described previously by Nadira et al. [37]. All the spots exhibiting differential accumulation with more than 1.5 fold between treatments and control were selected for statistical analysis. Statistical significance was resolved by Student’s *t* test (*p* < 0.05). Proteins with significant and reproducible changes were considered to be differentially expressed.

### 2.5. MALDI-TOF/TOF-MS and Protein Identification

MALDI-TOF/TOF MS analysis and protein identification of the tryptic-digested peptide mass fingerprint were evaluated using a MALDI-TOF/TOF mass spectrometer (ABI4700 System, CA, USA) according to Nadira et al. [37]. Target peptides with a MASCOT (Mascot is a software search engine) score more than 72 or a best ion score, based on both MS and MS/MS spectra, were considered to be significant (*p* < 0.05) candidate proteins. The Gene Ontology website (http://www.geneontology.org/) was used to identify the biological function.

### 2.6. Semiquantitative and Quantitative Real-Time PCR

To validate transcription in both the normal and treatment libraries, we used semiquantitative RT–PCR. PCR primers were designed according to the barley annotations (Table S1). For transcript amplification, 32 cycles were used, and 30 cycles were used for reference gene *HvTubulin* amplification. Total RNA was extracted using the RNeasy plant mini kit (Qiagen, Hilden, Germany) and reverse transcribed using the SuperScript III first-strand synthesis system (Axygen Biosciences, Union, CA, USA) according to the manufacturer’s instructions. qRT–PCR was performed using gene-specific primers. The PCR conditions were set according to He et al. [38]. The fold changes in the expression level relative to the control were expressed as 2^−ΔΔCt^. Barley *HvACTIN* was used as an internal control. The primers used for qPCR are presented in Table S1.

### 2.7. Isolation and Sequencing of S-Adenosylmethionine Synthetase 3 (HvSAMS3) of XZ5

First-strand cDNA was synthesized according to Kim [39] from total RNA using a RevertAid™ (Thermo Scientific, Waltham, MA, USA). For PCR amplification, the first Strand cDNA Synthesis Kit (Thermo Scientific, Waltham, MA, USA) was used as a template. PCR amplification of *HvSAMS3* was performed using LA-Taq™ DNA Polymerase (Takara, Beijing, China). The amplified PCR fragments were recovered, cloned into the pMD19-T vector using a TA cloning kit (Takara, Beijing, China), and sequenced. The primers to clone *HvSAMS3* are presented in Table S2.

### 2.8. Bioinformatics Analysis

Database searches for similarity were achieved using the GenBank database and the Nucleotide-nucleotide BLAST (BLASTN) and Nucleotide 6-frame translation-protein (BLASTX) algorithms. The sequences were translated to identify open reading frames using the ORF Finder in the National Center for Biotechnology Information (NCBI) database (http://www.ncbi.nlm.nih.gov/gorf/gorf.html). Multiple sequence alignments of the encoded full-length SAMS3 proteins from representative plant species were aligned using the clustalX 2.01 program [40]. To gain insight into the phylogenetic relationships and functional associations of *SAMS* genes, 42 plant *SAMSs* from nine monocots and one dicot plants were used to construct a phylogenetic tree based on maximum-likelihood (ML) methods using MEGA 5.0 [41]. The gene name and locus ID for these 10 *SAMSs* family genes are provided in Table S3.

The amino acid sequences of four *Arabidopsis SAMS* genes were used as the query to search the barley genome in the NCBI database (http://www.ncbi.nlm.nih.gov/) using the Protein-protein BLAST (BLASTP) program (https://blast.ncbi.nlm.nih.gov/Blast.cgi?PAGE=Proteins). Based on domain composition analyses using the Pfam (http://pfam.sanger.ac.uk/) and NCBI CDD (http://www.ncbi.nlm.nih.gov/cdd) programs, 4 *SAMS* sequences were identified in the barley genome.

### 2.9. Subcellular and Tissue Localization of HvSAMS3

The ORF of *HvSAMS3* of XZ5 was directly amplified from the full-length cDNA using *HvSAMS3*-specific primers (Table S2) and was cloned into the binary vector pCAMBIA 1300 containing a CaMV 35S promoter:GFP cassette to create the HvSAMS3–GFP (Green fluorescent protein) fusion protein according to He et al. [38]. The construct was familiarized into onion epidermal cells using particle bombardment. The fusion protein was transiently expressed in onion epidermal cells. The subcellular localization of the fusion protein was observed using a confocal microscope (LSM780; Carl Zeiss, Oberkochen, Germany). GFP fluorescence, the bright field image, and the red fluorescence (RFP plasma membrane marker) were imaged simultaneously and merged. All the transient expression assays were repeated at least three times. Tissue-specific expression analysis of *HvSAMS3* from XZ5 was carried out in three replicates using seedlings grown in soil for 25 d and seeds (five seeds per replicate). RNA extraction, reverse transcription, and RT–PCR were performed as described above. The primers are shown in Table S2.

### 2.10. BSMV Inoculation and Treatment

BSMV analysis was conducted according to He et al. [38]. RT-PCR was performed using primers containing NheI sites (Table S2) to obtain a 286-bp cDNA fragment of the barley phytoene desaturase gene (*HvPDS*) and a 254-bp cDNA fragment of *HvSAMS3*. The inoculation was conducted on the second leaf of XZ5 plants at the two-leaf stage. For BSMV:HvPDS VIGS, the leaf samples were chosen based on the observed phenotype, and photos of the leaf were acquired using a camera (EOS 7D, Canon, Japan). To confirm the VIGS effectiveness and function of *HvSAMS3* in XZ5, eight independent sets of treatments were performed: mock-inoculated with BSMV:γ; mock-inoculated with BSMV:γ and treated with drought, salinity, and D+S; BSMV:HvSAMS3-inoculated seedlings; and BSMV:HvSAMS3-inoculated seedlings treated with drought, salinity, and D+S. Every treatment contained 5 biological replicates, and each had 5 plants. Additionally, the transcript levels of *HvPDS* and *HvSAMS3* were measured using quantitative RT-PCR and semiquantitative RT-PCR, respectively. Ten days after the treatments, the second leaves from the upper of barley seedlings were harvested for measurements of the following parameters: potassium (K^+^) concentration; endogenous polyamide (PA) and 1-aminocyclopropane-1-carboxylic acid (ACC) (ethylene) contents; polyamine oxidase (PAO), and diamine oxidase (DAO), and antioxidant enzyme activities.

### 2.11. Ethylene and Biotin Treatments and Gene Function Analysis

The significance of ethylene and biotin synthesis and utilization in the plant cell and its potential importance in the regulation of ethylene and biotin metabolism are involved in the SAM cycle. Here, to verify the function and role of ethylene and biotin of *HvSAMS3* in XZ5, eight treatments were performed: (1) BSMV:γ mock-inoculated seedlings grown in soil for 25 d (nonsalinized), in which soils in the pots were kept humid (60–80% water-holding capacity); (2) BSMV:γ mock-inoculated seedlings were grown in soil for 15 d, then exposed to drought and salinity for 10 d with 200 mM NaCl and withholding the water supply; (3) BSMV:HvSAMS3-inoculated seedlings grown in soil for 25 d, in which soils in the pots were kept at water-holding capacity of 60–80%; (4) BSMV:HvSAMS3-inoculated seedlings were grown in soil for 15 d, and then drought and salinity were applied for 10 d with 200 mM NaCl and withholding the water supply; (5) BSMV:HvSAMS3-inoculated seedlings grown in soil and sprayed with ethylene every alternate day from 16 d to 25 d; (6) BSMV:HvSAMS3-inoculated seedlings grown in soil for 15 d and then exposed to D+S for 10 d by withholding the water supply coupled with ethylene (150 µM) spray every alternate day from 16 d to 25 d; (7) BSMV:HvSAMS3-inoculated seedlings grown in soil and sprayed with biotin every alternate day from 16 d to 25 d; (8) BSMV:HvSAMS3-inoculated seedlings grown in soil for 15 d and then exposed to D+S for 10 d by withholding the water supply coupled with biotin (200 nM) spray every alternate day from 16 d to 25 d. The ethylene-sprayed pot was kept in a separated chamber. After treatment, the phenotypes of all plants were investigated and recorded. Finally, six individuals were randomly selected from each treatment to assess the physiological indexes.

### 2.12. Leaf Dry Weight and Potassium (K^+^) Concentration Measurement

The leaves of BSMV-inoculated and ethylene- and biotin-treated plants were collected and dried at 80 °C for 72 h. Then, the dried leaf samples were powdered and weighed and then were ashed at 550 °C for 12 h. The ash was digested with 5 mL of 30% HNO_3_ and then was diluted using deionized water [42]. The K^+^ concentrations were determined using flame atomic absorption spectrometry (Shimadzu, AA-6300, Kyoto, Japan).

### 2.13. PA Contents, H_2_O_2_ Content, Biotin Concentration, PAO, and DAO Activities and Antioxidant Enzyme Activity

The endogenous content of the PAs putrescine (Put), spermidine (Spd), and spermine (Spm) was determined as described in Choudhary et al. [43]. Polyamine oxidase (PAO) and diamine oxidase (DAO) were measured according to Filippou et al. [44]. The plant biotin concentration was determined using an ELISA kit (Becton, Dickinson and Company, Franklin Lakes, NJ, USA). Superoxide dismutase (SOD, EC 1.15.1.1), catalase (CAT, EC 1.11.1.6), and peroxidase (POD, EC 1.11.1.7) activities were determined according to Wu et al. [36], and ascorbate peroxidase (APX, EC 1.11.1.11) activities were determined according to Chen et al. [16]. H_2_O_2_ in the leaf was extracted, and the content was measured spectrophotometrically as previously described by Ahmed et al. [35].

### 2.14. Gas Chromatography

Ethylene production in the leaves of barley was measured as described by Cao et al. [45]. The fresh leaf tissues (0.5 g fresh weight) were immediately weighed after collection and then were placed in sealed glass vials containing water-saturated filter paper. The samples were incubated for 1 h under illumination. One milliliter of gas was collected using a gas-tight syringe and injected into a gas chromatograph (Focus GC; Thermo Fisher Scientific, MA, USA) equipped with a capillary column (CP-carboPLOT P7; Varian, CA, USA) and a flame ionization detector for ethylene determination. Ethylene production was calculated based on known ethylene standards and the leaf fresh weight.

### 2.15. Statistical Analysis

All the data are presented as mean values. Analysis of variance (ANOVA) was conducted to determine differences between the treatments. The significance of the differences between the Tibetan wild and cultivated barley or between the control and treatments was evaluated using LSD (Least Significant Difference) multiple range tests (P < 0.05) in SAS 9.2 statistical software (SAS Institute Inc., Cary, NC, USA).

## 3. Results

### 3.1. Ultrastructure of Leaf Chloroplast and Root Tip Cells in Response to Combined Drought and Salinity Stresses

The deterioration of chloroplastic and mitochondrial ultrastructure caused by drought and combined stress was more prevalent in CM72 than in the two Tibetan wild barley genotypes (Figure S1A). Electron micrographs of meristematic cells from drought- and salinity-stressed barley seedlings revealed obvious ultrastructural changes (Figure S1B). The meristematic cells were clearly damaged in combined stressed plants, with injuries being more pronounced in CM72.

### 3.2. Differential Abundant Proteins Induced by Drought and Salinity in Leaves

**Figure 1:**
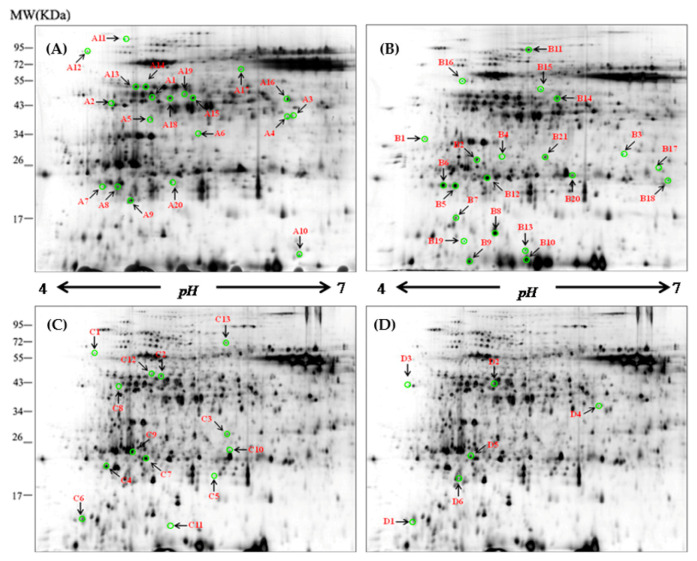
Representative two-dimensional gel electrophoresis (2-DE) maps of barley leaf proteins in XZ5. The proteins were isolated from the leaf of XZ5 exposed to drought (**A**), salinity alone (**B**), and combined stress D+S [(D+S vs. D (**C**), D+S vs. S (**D**)] during the vegetative stage at the 4% soil moisture level. Each 150-µg protein sample in 0.8% (v/v) Immobilized pH Gradient (IPG) buffer (Amersham Biosciences) was loaded onto analytical gels for 2-D gel image analysis using the silver-staining method. The spots were visualized by silver staining. Differentially accumulated protein spots are indicated by green sashes. Arrows indicate the differentially expressed protein spots whose expression levels were significantly induced (fold change ≥ 1.5) or downregulated (fold change < −1.50) in XZ5. The numbered leaf protein spots were labeled A1–A20, B1–B21, C1–C13, and D1–D6.

**Figure 2:**
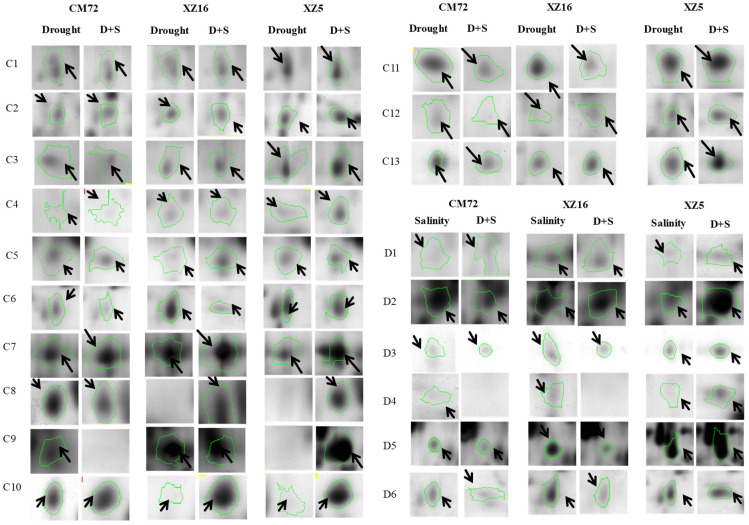
‘Spot view’ of the abundance of differentially expressed proteins (indicated with green circles) in the leaves of three barley genotypes XZ5, XZ16 and CM72 under drought stress, and salinity stress (200 mM NaCl) and D+S stress conditions. Protein spot IDs refer to numbers in Figure 1C,D and Table 1 and Table 2.

The total proteins from leaves were resolved into more than 1200 spots (ranging from 1250 to 2015) in each of the 2-DE gels. There was a differential accumulation of proteins under different treatments (Tables S4 and S5; Figure S2A,B). Further comparison of the genotypic differences revealed differentially abundant proteins (DAPs) under drought, salinity, and combined drought and salinity treatments using MALDI-TOF/TOF MS (Figure 1). Sixty protein spots were related to drought and salinity tolerance, being significantly increased by drought (drought versus control, fold change >1.50), salinity (salinity versus control) and combined drought and salinity (D+S versus drought and D+S versus salinity) in XZ5, but unchanged/decreased in XZ16 or unchanged in XZ5 but decreased in CM72 (Figure 1). Therefore, these 60 protein spots were selected for MALDI-TOF/TOF MS analysis. Details of the expression pattern of drought, salinity alone, and in combination are shown in Figure 2, Figure S3A,B).

The 60 identified proteins were classified into eight major categories according to Bevan et al. [46] (Table 1 and Table 2, Tables S6–S8, and Figure S4A) (http://www.peptideatlas.org/PASS/PASS01597 Dataset tag: barley20200616). Among the identified 60 DAPs, 20 (drought versus control, spots A1 to A20), 21 (salinity versus control, B1 to B21), 13 (D+S versus drought, C1 to C13) and 6 (D+S versus salinity, D1 to D6) DAPs were treatment specific (Table 1 and Table 2; Tables S6–S8). These included S-adenosylmethionine synthetase 3 (SAMS3), phosphoribulokinase, ribulose bisphosphate carboxylase, and elongation factor Tu. SAM3 (D2) was exclusively expressed or upregulated by the combined stress of drought and salinity (Table 2, Figure 2 and Figure S4B).

### 3.3. Cloning of the HvSAMS3 and Bioinformatics Analysis

**Figure 3:**
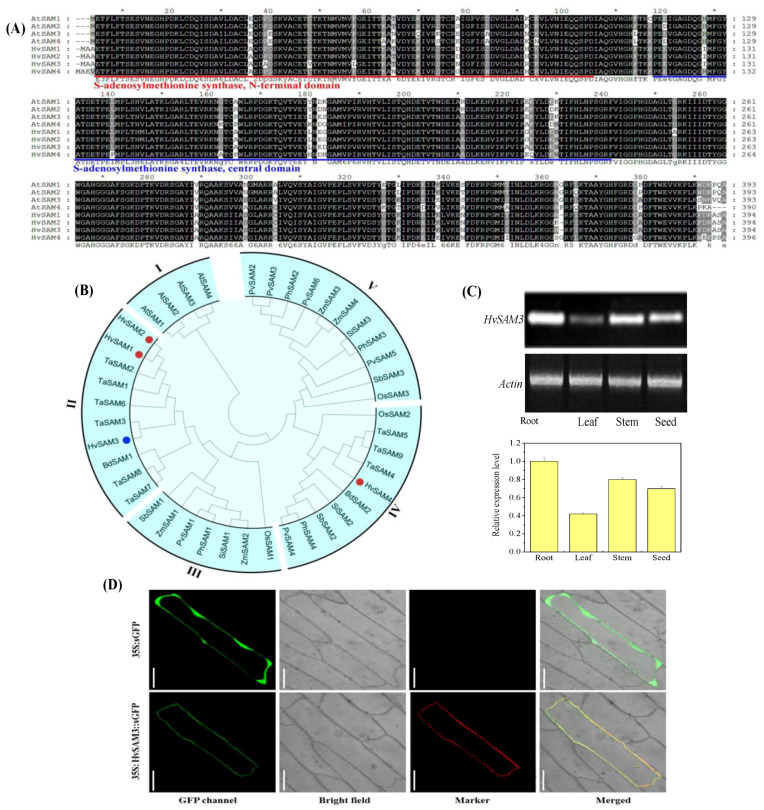
Identification of S-adenosylmethionine synthetase (SAM) proteins in monocots. (**A**) Multiple alignment of amino acid sequences of *AtSAM1–AtSAM4* and identification of SAM in barley (*HvSAMS1–HvSAMS4*). The conserved S-adenosylmethionine synthetase N-terminal domain and S-adenosylmethionine synthetase central domain are shown. (**B**) Phylogenetic analysis of SAM proteins in monocots. The colored dots symbolize SAM members in barley. Tissue expression pattern and subcellular localization of S-adenosylmethionine Synthetase 3 (HvSAMS3). (**C**) RT-PCR analysis of the relative transcript levels of *HvSAMS3* in different tissues of XZ5. (**D**) Transient expression of GFP (Green fluorescent protein) and the *HvSAMS3*-sGFP fusion protein in onion epidermis cells. Images are GFP fluorescence (GFP; green pseudocolor), red fluorescence [RFP PM marker (plasma membrane-localized marker); red pseudocolor], optical photomicrographs (bright field), and merged (optical photomicrographs, RFP PM marker, and GFP fluorescence). The data shown are representative of three independent experiments (*n* = 3). Scale bars, 100 μ.

The protein SAMS3 (Spot D2) was obtained from 2-DE and identified by MALDI-TOF/TOF MS, and the corresponding genes were also found in GenBank (Accession no. AM039895.1). Here, we cloned *HvSAMS3* from XZ5 to determine its biological function. Sequence analysis from XZ5 revealed that *HvSAMS3* cDNA comprises a putative open reading frame (ORF) of 1185 bp encoding *HvSAMS3* of 393 amino acids with the calculated molecular weight of 43.1 kDa and pI of 5.51 (Figure S4C). The amino acid sequence analysis of the deduced protein HvSAMSs revealed high sequence identity to SAM synthetases of other plant species, such as *Arabidopsis thaliana* (98%) and *Oryza sativa* (99%). The four *SAMS* were named *HvSAMS1* (AM039893), *HvSAMS2* (AM039894), *HvSAMS3* (AM039895.1), and *HvSAMS4* (AM039896) according to their phylogenetic relationship with the four *Arabidopsis AtSAMSs*. These SAM proteins possessed a SAM N-terminal domain and a SAM central domain (Figure 3A and Figure S5).

Phylogenetic analysis indicated that the 42 SAM proteins from 10 plant species clustered into five main groups (groups I–V) with strong bootstrap support (Figure 3B). Intriguingly, all four SAMs from the dicot plant (*Arabidopsis*) were clustered into group I, while the remaining 38 SAMs from the monocot species were combined into the other four groups (I, II, IV, and V). *HvSAMs* are closely related to *TaSAMs* and are distributed in the same groups (group II and group IV), while group II corresponds to the *HvSAMS3*. *HvSAMS1*, *2* and *3* were clustered into group II, together with *TaSAMS1*, *2, 3, 6, 7*, and *8*, and *BdSAMS1*. Additionally, *HvSAMS4* was located in group IV with *TaSAMS4*, *5* and *9*, *BdSAMS2, SiSAMS2, SbSAMS2, PhSAMS4, PvSAMS4,* and *OsSAM2*. These results indicated that SAM genes are highly conserved in flowering plants, and *HvSAMS3* homologs are predicted to play similar functions in monocot species.

### 3.4. Expression and Subcellular Localization of HvSAMS3

Tissue-specific expression of the *HvSAMS3* gene in the root, stem, leaf, and grain of the XZ5 genotype was examined by RT-PCR (Figure 3C). The *HvSAMS3* transcript was found to be preferentially accumulated in the root and stem of XZ5, and comparatively lower levels of expression were detected in the leaf and grain. To investigate the subcellular localization of *HvSAMS3*, the GFP reporter gene transnationally fused to the *HvSAMS3* coding region was used in a transient assay in onion epidermis cells. The fused green fluorescence overlapped completely with the RFP red fluorescence of a marker protein, pm-rb CD3-1008 [47], which was specifically located on the cell membrane in onion (Figure 3D). The HvSAMS3-GFP protein was detected mainly in the cell membrane, while the control (transformation of GFP construct) was observed in the entire region of the cell membrane (Figure 3D).

### 3.5. Disruption of HvSAMS3 Reduces Plant Growth and Stress Tolerance in XZ5

**Figure 4:**
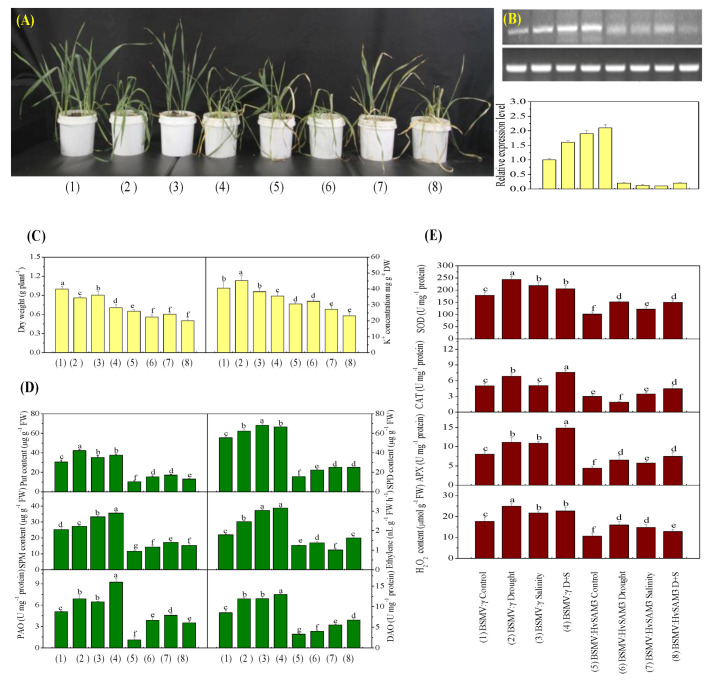
Functional assessment of *HvSAMS3* in wild barley XZ5 via barley stripe mosaic virus-based virus-induced gene silencing (BSMV-VIGS). (**A**) Photography was performed 10 days after treatment. (**B**) RT-PCR analysis of the relative transcript levels of *HvSAMS3* in XZ5 leaves. (**C**) Dry weight and K^+^ concentration in the roots of mock and BSMV:HvSAMS3-inoculated XZ5 seedlings. (**D**) Levels of putrescine (Put), spermidine (Spd) and spermine (Spm), ethylene production, and activities of polyamine oxidase (PAO) and diamine oxidase (DAO). (**E**) Superoxide dismutase (SOD), catalase (CAT), ascorbate-peroxidase (APX) activities and H_2_O_2_ content in response to drought or salinity alone and combined stresses of drought and salinity in BSMV:HvSAMS3-inoculated XZ5 seedlings compared with the BSMV:γ mock-inoculated seedlings. Error bars represent SD values (*n* = 4). Different letters indicate significant differences (*p* < 0.05) among the treatments. (1) BSMV:γ mock-inoculated seedlings grown in soil for 25 d (nonsalinized; 60–80% water-holding capacity); (2) BSMV:γ mock-inoculated seedlings grown in soil for 15 d and then exposed to drought for 10 d by withholding the water supply; (3) BSMV:γ mock-inoculated seedlings grown in soil for 15 d and then exposed to salinity for 10 d (200 mM NaCl; 60–80% water-holding capacity); (4) BSMV:γ mock-inoculated seedlings grown in soil for 15 d and then exposed to drought and salinity for 10 d (200 mM NaCl; withholding the water supply); (5) BSMV:HvSAMS3-inoculated seedlings grown in soil for 25 d (60–80% water-holding capacity); (6) BSMV:HvSAMS3-inoculated seedlings grown in soil for 15 d and then exposed to drought for 10 d by withholding the water supply; (7) BSMV:HvSAMS3-inoculated seedlings grown in soil for 15 d and then exposed to salinity for 10 d (200 mM NaCl, 60–80% water-holding capacity); (8) BSMV:HvSAMS3-inoculated seedlings grown in soil for 15 d and then exposed to drought and salinity for 10 d (200 mM NaCl and withholding the water supply).

The BSMV-VIGS-based gene silencing technique was employed to determine whether the disruption of *HvSAMS3* at the mRNA level affects plant growth in XZ5. Approximately 90% of the *HvPDS* transcripts were suppressed in the BSMV:HvPDS-inoculated plants compared with those in the control (Figure S6A,B). Thus, the BSMV-VIGS system demonstrated potential effects of *HvSAMS3* after the silencing of its expression (Figure 4). BSMV:HvSAMS3-inoculated seedlings under the control and drought, salinity, and D+S treatments showed decreased plant growth and tolerance compared with the mock-inoculated barley plants (BSMV-γ) at 20 dpi (Figure 4A), when the visual symptoms of photo-bleaching were observed in the most BSMV:HvPDS-inoculated plants. As shown in Figure 4B, the transcript levels of *HvSAMS3* in the silenced plants were significantly reduced, leading to a silencing efficiency of 75% in standard VIGS experiments compared with those in the mock-infiltrated plants. By contrast, the transcript levels of *HvSAMS1, HvSAMS2*, and *HvSAMS4* in *HvSAMS3*-silenced plants varied in different stress treatments and were comparable to the levels in the mock-infiltrated control plants (Figure S6C). Additionally, compared with the mock control, the dry weight and K^+^ concentration in the leaves of the BSMV:HvSAMS3-inoculated seedlings were decreased under the control, drought, salinity, and combined stress treatments (Figure 4C).

### 3.6. HvSAMS3 Regulates Polyamine Synthesis and Antioxidant Enzyme Activities

The silencing of *HvSAMS3* significantly decreased the Spd, Spm, Put, and ethylene levels, as well as the PAO and DAO activities, in leaves under both normal and stress conditions (Figure 4D). The Spd, Spm, and Put levels, as well as the PAO and DAO activities, of the BSMV:HvSAMS3-inoculated XZ5 seedlings were decreased under all three treatments compared with the mock-inoculated XZ5 seedlings. Additionally, ethylene emissions in the leaf of the mock-inoculated XZ5 seedlings were induced by all three treatments (Figure 4D). However, the ethylene emissions of the BSMV:HvSAMS3-inoculated XZ5 seedlings were decreased under all three treatments compared with those of the mock-inoculated barley plants (Figure 4D). Furthermore, *HvSAMS3*-silencing decreased the SOD, CAT, and APX activities and H_2_O_2_ content (Figure 4E). The SOD, CAT, and APX activities and H_2_O_2_ content were induced by all three treatments in the mock-inoculated barley plants (BSMV:γ), but lower activities were found in the BSMV:HvSAMS3-inoculated plants. Among the three stresses, the mean fold induction of SOD and APX activities were 35% and 46% in the mock-inoculated barley plants (BSMV:γ), respectively, but they were only 11% and 15%, respectively in the BSMV:HvSAMS3-inoculated plants (Figure 4E). Although drought stress induced CAT activity by 35% in BSMV:γ plants, significantly lower activity (18–22%) was observed in BSMV:HvSAMS3-inoculated plants than in their respective controls (Figure 4E). CAT activity showed no significant difference after salt stress in BSMV:γ- and BSMV:HvSAMS3-inoculated plants, while CAT activity was increased in both inoculated plants under combined stress compared with their respective controls (Figure 4E). Among the three stresses, the mean fold induction of the H_2_O_2_ content was 38% in the mock-inoculated barley plants (BSMV:γ) but was only 15% in BSMV:HvSAMS3-inoculated plants (Figure 4E).

### 3.7. Effects of Exogenous Ethylene and Biotin

**Figure 5:**
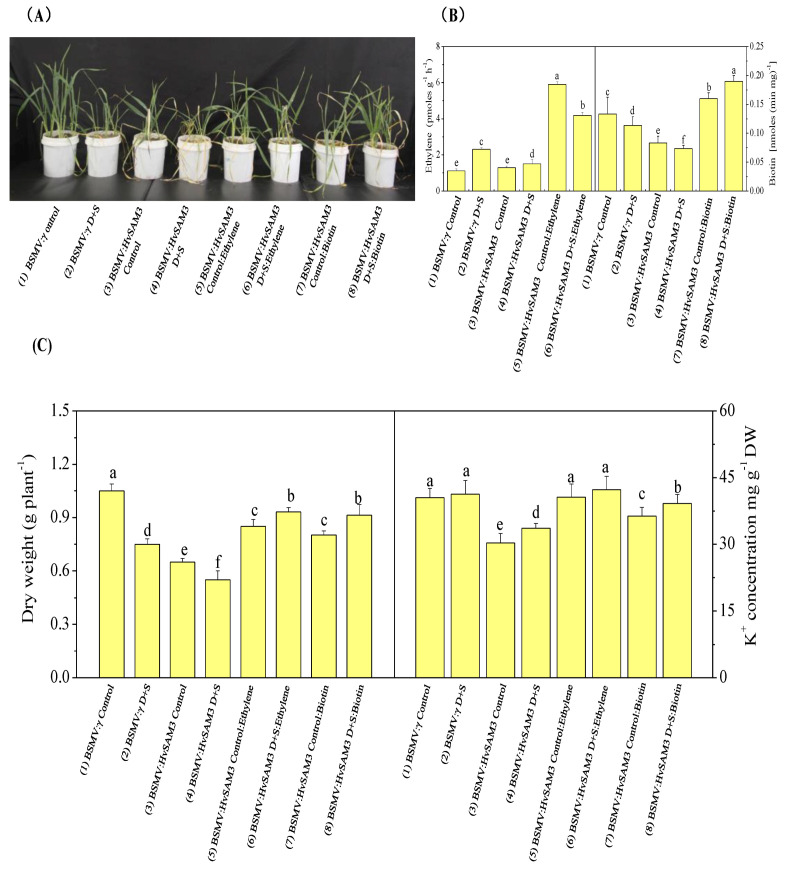
Functional assessment of *HvSAMS3* in wild barley XZ5 via BSMV-VIGS. (**A**) Photography was performed at 10 days after treatment. (**B**) Ethylene production and biotin concentration of mock and BSMV:HvSAMS3-inoculated XZ5 seedlings. (**C**) Root dry weight and root K^+^ concentration of mock and BSMV:HvSAMS3-inoculated XZ5 seedlings. Error bars represent SD values (*n* = 4). Different letters indicate significant differences (*p* < 0.05) among the treatments. (1) BSMV:γ mock-inoculated seedlings grown in soil for 25 d (nonsalinized), in which the soils in the pots were kept humid (60–80% water-holding capacity); (2) BSMV:γ mock-inoculated seedlings grown in soil for 15 d and then exposed to drought and salinity for 10 d with 200 mM NaCl and withholding the water supply; (3) BSMV:HvSAMS3-inoculated seedlings grown in soil for 25 d, in which the soils in the pots were kept humid (60–80% water-holding capacity); (4) BSMV:HvSAMS3-inoculated seedlings grown in soil for 15 d and then exposed to drought and salinity for 10 d with 200 mM NaCl and withholding the water supply; (5) BSMV:HvSAMS3-inoculated seedlings grown in soil and sprayed with ethylene every alternate days from day 16 to day 25; (6) BSMV:HvSAMS3-inoculated seedlings grown in soil for 15 d and then exposed to D+S for 10 d by withholding the water supply coupled with ethylene spraying every alternate day from 16 d to 25 d; (7) BSMV:HvSAMS3-inoculated seedlings grown in soil and sprayed with biotin every alternate day from 16 d to 25 d; (8) BSMV:HvSAMS3-inoculated seedlings grown in soil for 15 d and then exposed to D+S for 10 d by withholding the water supply coupled with biotin spraying every alternate day from 16 d to 25 d.

We investigated the role of *HvSAMS3* in drought and salinity tolerance concerning the synthesis and degradation of ethylene and biotin in the plant cell (Figure 5). Exogenous application of ethylene (150 µM) and biotin (200 nM) improved the growth and development of BSMV:HvSAMS3 plants compared with those of the control (Figure 5A). Combined drought and salinity-treated mock-inoculated barley plants showed elevated ethylene levels compared with those of the control. The ethylene content of BSMV:HvSAMS3-inoculated barley plants was slightly increased under combined drought and salinity stress, but the difference was not significant; however, the levels were decreased compared with those of BSMV:γ D+S-inoculated barley plants (Figure 5B). In our experiment, exogenous ethylene application on stressed plants significantly increased endogenous ethylene production, but its incremental magnitude was lower in BSMV:HvSAMS3-inoculated plants under combined stress compared with the respective control (Figure 5B). By contrast, biotin production was decreased in both types of inoculated plants. However, an exogenous biotin supplementation of stressed plants upregulated endogenous biotin production, but the degree of increment was higher in BSMV:HvSAMS3-inoculated plants under combined stress than in control plants. Compared with the mock-inoculated barley plants (BSMV:γ), the dry weight and K^+^ concentration were significantly reduced in BSMV:HvSAMS3-inoculated plants (Figure 5C). However, the application of ethylene and biotin to stressed plants increased the dry weight and K^+^ concentration in the leaves of the BSMV:HvSAMS3-inoculated seedlings compared with those in the control plants (Figure 5C).

## 4. Discussion

Drought and soil salinity stresses co-occur in the natural environment, especially in dry hot valleys. Improving our understanding of the plant response to drought and salinity stresses is an important research priority. To identify important stress related proteins and key genes to understand the underlying mechanisms in stress tolerance, we studied the cellular, proteomics, and molecular responses of the Tibetan wild and cultivated barley by imposing combined salinity and drought stresses.

### 4.1. The Identified Proteins Play an Important Role in Tolerance to the Combined Stresses of Drought and Salinity

**Figure 6:**
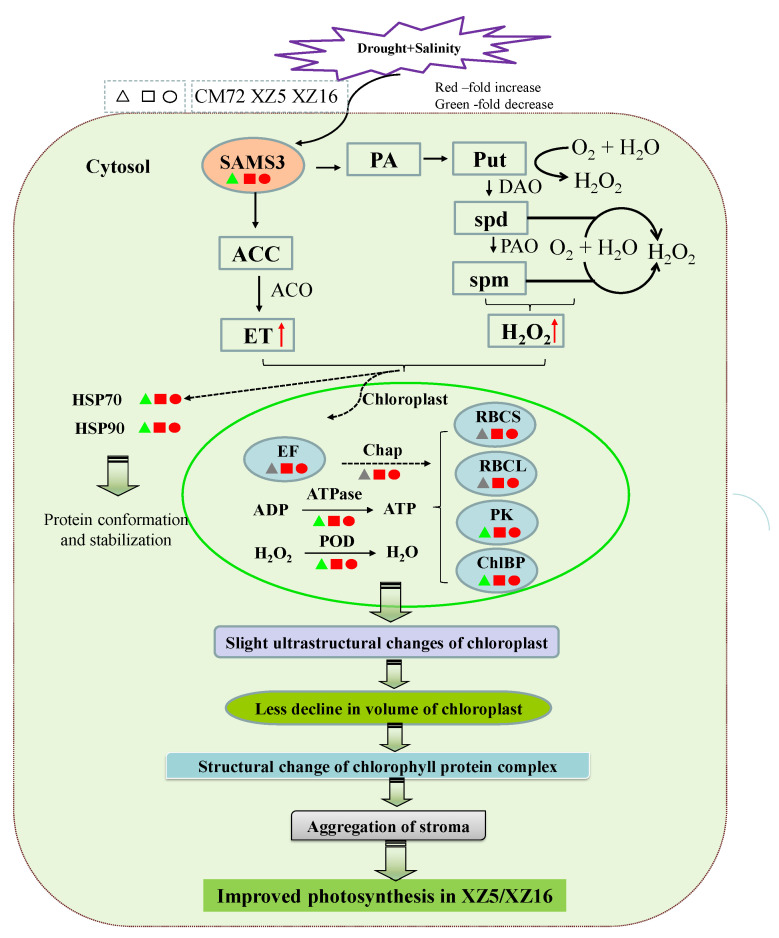
Integrated schematic of the mechanisms involved in the tolerance and adaptation of combined drought and salinity stress in Tibetan wild barley. The proteins in red, gray, and green are upregulated, nonchanged, and downregulated according to Table 1 and Table 2, respectively. The red arrows indicate upregulated changes. ACC, S-aminocyclopropane-1-carboxylate; ACO, S-aminocyclopropane-1-carboxylate oxidase; ATPase, ATP synthase subunit beta; Chap, 20 kDa chaperonin; ChlBP, chlorophyll a-b binding protein 1; DAO, diamine oxidase; ET, ethylene; EF, elongation factor Tu; HSP, heat shock protein; PA, polyamine; PAO, polyamine oxidase; PK, phosphoribulokinase; POD, peroxiredoxin-2E-2; Put, putrescine; RBCL, ribulose bisphosphate carboxylase large chain; RBCS, ribulose bisphosphate carboxylase small chain clone 512; SAM, S-adenosyl-L-methionine synthetase; spd, spermidine; spm, spermine. Moreover, slight ultrastructural changes in chloroplasts lead to a lower chloroplast volume, aggregation of stroma, structural changes in chlorophyll protein complex, and improved ATP synthesis.

Significant differences in protein abundance under the combined stresses of drought and salinity were found during proteome analysis. Based on the experimental results and identified proteins related to the tolerance of combined drought and salinity stresses in this study, we propose the mechanism involved in combined stress tolerance (Figure 6). The mentioned proteins were upregulated in the combined stressed leaves of XZ5 and XZ16 but downregulated or remained unaltered in CM72 compared with those under drought or salinity stress alone (Figure 6, Table 1 and Table 2).

Environmental stresses, such as temperature, drought, and salinity, significantly affect photosynthesis in plants [48,49]. Proteins whose abundance depends significantly on drought and salinity are mostly associated with photosynthesis [50]. For example, the levels of ribulose bisphosphate carboxylase small chain (C3), ribulose bisphosphate carboxylase large chain (C5), phosphoribulokinase (C8), and chlorophyll a-b binding protein (C9) were remarkably enhanced in XZ5 and XZ16 compared with those in CM72 (Figure 6, Table 1), indicating that stressed barley plants maintain the light reaction proteins to permit the sufficient transfer of excitation energy. Similar results were observed in a previous report on mulberry leaves under salinity- and drought-stressed conditions [51]. ATP synthase subunit β (C1) and peroxiredoxin-2E-2 (POD, D1) also showed higher expression levels in Tibetan wild barley than CM72 (Figure 6, Table 1). The results suggest that in XZ5 and XZ16, higher expression of ATP synthase and POD activity occur under combined stress. This phenomenon occurred by elevating the capacity of scavenging combined stress-induced peroxide over accumulation and higher ATP supply to meet the higher energy demand in chloroplasts due to stress. ATP synthase upregulation has also been observed in wheat [52]. Another important effect that improved plant growth and the photosynthetic abilities of the plants is the loss of balance between the production of reactive oxygen species and antioxidant defense, causing slight ultrastructural changes in chloroplasts in XZ5 (Figure S1), leading to a lower decline in the volume of chloroplasts, aggregation of stroma, structural changes in the chlorophyll protein complex, and improved ATP synthesis (Figure 6). Additionally, the roles of PAs in plant growth and development and the mechanisms underlying their function can be explored by studying the relationship between PA metabolism and plant hormones, as well as the effects of PA metabolism on plant signaling substances (Figure 6). The metabolism of PAs in plants is closely connected to many other metabolic pathways. The H_2_O_2_ produced by PA oxidation functions in the signal transduction process of plants during abiotic stress responses, and it affects stomatal closure induced by abscisic acid (ABA) [53]. In our present and previous study, XZ5 showed a lower accumulation of H_2_O_2_, confirming that Tibetan wild barley shows less oxidative damage, which is a phenomenon that is potentially linked to plant recovery from stress (Figure 6). Additionally, the expression of elongation factor Tu (C2) was increased in all genotypes (with higher increase in XZ5) under D+S stress over drought stress (Figure 6 and Table 1).

Higher protein synthesis is important to restore the damaged proteins for effective plant cell metabolic activity and general growth. Increased protein synthesis may be the key factor regulating the tolerance and adaptation mechanisms of combined drought and salinity stress in Tibetan wild barley. In rice, proteomic analyses of cold and heat stress responses identified plastid elongation factor Tu as an upregulated protein [54,55]. Wang et al. [56] reported that normal protein conformation and, thus, cellular homeostasis was maintained by heat shock proteins in the plant stress defense system. Heat shock proteins are responsible for the functions of protein assembly, folding, translocation, and degradation in many cellular processes, as well as the stabilization of membranes, proteins, and protein refolding under stress conditions. These proteins often assist the folding of de novo synthesized polypeptides and the import/translocation of precursor proteins [56]. Under salinity stress conditions, HSPs accumulate in alfalfa (*Medicago sativa* L.) leaves, which is a finding that corroborates our present results. [57]. The increased heat shock protein levels indicate their role in combined stress tolerance in barley in the present study. Comprehensive studies on the post-translational modification of heat shock proteins help to elucidate the mechanisms involved in the D+S stress tolerance of XZ5 and XZ16. Additionally, peroxiredoxin-2E-2 proteins (D1) were upregulated in stressed XZ5 and XZ16 leaves. The higher accumulation of these proteins indicates that Tibetan wild barley plant cells initiate their antioxidant mechanisms to maintain redox homeostasis and resist combined stresses. The protein chalcone-flavonone isomerase (D5), which is involved in the production of flavanols and the regulation of secondary metabolism, was upregulated in XZ5. These results suggest that in response to combined stress, the biosynthesis of this secondary metabolite occurs in Tibetan wild barley.

### 4.2. Ethylene, Biotin, Polyamines, and Antioxidant Enzymes Involved in HvSAMS3-Mediated Tolerance to Combined Stress

**Figure 7:**
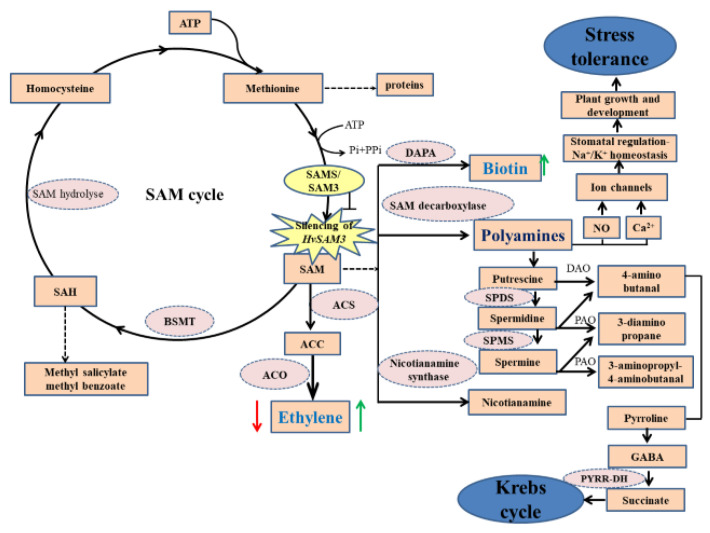
A proposed model for *HvSAMS3* improvement of plant growth and development regulated by biotin and ethylene in wild barley XZ5. *HvSAMS3* silencing of SAM cycle involving in ethylene, polyamines, and biotin biosynthesis process in Tibetan barley plants. Silencing of *HvSAMS3* decreases ethylene, putrescine, spermidine and spermine, polyamine oxidase, and antioxidant enzyme activities, whereas these effects were alleviated by exogenous ethylene and biotin under combined stress. Thick arrows indicate up- and downregulated changes. Dashed arrows indicate multiple steps, the blunted arrow indicates the silence effect of *HvSAMS3*. Thick red arrows mark the effect of the BSMV:HvSAMS3, and thick green arrows mark relative changes by exogenous ethylene and biotin under combined drought and salinity stress. SAM: S-Adenosyl-L-methionine; SAMS: S-Adenosyl-L-methionine synthase; ACC: S-Aminocyclopropane-1-carboxylate; ACS: S-Aminocyclopropane-1-carboxylate synthase; ACO: S-Aminocyclopropane-1-carboxylate oxidase; BSMT: benzoic acid/salicylic acid; DAO: Diaminoxidase; DAPA: 8-diamino pelargonic acid aminotransferase; GABA: Aminobutyric acid; NO: Nitric oxide; PAO: polyamine oxidase; PYRR-DH, pyrroline dehydrogenase; SAH: S-Adenosyl-L-homocysteine; SAHH: S-Adenosyl-L-homocysteine hydrolase; SPDS: Spermidine synthase; SPMS: Spermine synthase.

SAMSs work as an aminopropyl and methyl donors for ethylene and PAs [58,59]. Additionally, ethylene and PAs are involved in the response to biotic and abiotic stresses in plants [60]. This function confirms the important role of SAMs in Spd and Spm biosynthesis for stress response in plants [61]. In the present study, *SAMS3* was upregulated in XZ5 and XZ16 and repressed in CM72 under D+S stress, suggesting that this protein might be involved in the tolerance to drought and salinity of XZ5. SAM in the PA biosynthetic pathway is also a precursor for ethylene synthesis (Figure 7), and studies have demonstrated that PA synthesis competes with ethylene synthesis. Additionally, the metabolism of PAs is related to the production of nitric oxide (NO), which is an essential signaling component for plant growth [62]. Tolerance to multiple abiotic stresses including drought, salinity, and cold is decreased in transgenic plants due to decreased levels of Spd and Spm associated with the downregulated transcripts of *Oryza sativa OsSAMDC2* and *Medicago sativa* subsp. falcata *MfSAMS1* [63,64]. Similarly, the downregulation of *SAMDC* expression results in reduced levels of Spd and Spm and decreased salinity tolerance of transgenic tobacco plants [65]. In the current study, biotin synthesis in the plant cell connotes intracellular trafficking of biotin and precursors, indicating transport mechanisms. However, the intracellular trafficking of biotin and intermediates is largely unknown, despite its potential importance in the control of metabolic fluxes within the cell [66].

Lower activities of PAO and antioxidant enzymes were observed in BSMV:HvSAMS3-inoculated plants. Compared with the induction in antioxidant enzyme activities in BSMV:γ-inoculated plants under drought and salinity, the lower levels of antioxidants in BSMV:HvSAMS3-inoculated plants were associated with decreased drought and salinity tolerance. PAO catalyzes the oxidation of Spd and Spm to produce H_2_O_2_ [67], while H_2_O_2_ is involved in signaling in plant adaptation to stress, including the induction of antioxidant enzyme-encoding genes [68]. Based on these results, lower levels of antioxidant enzyme activities in BSMV:HvSAMS3-inoculated plants may be the result of the reduced levels of Spd and Spm and PAO activities under drought and salinity (Figure 4D).

### 4.3. SAMS3 Contributes to Combined Stress Tolerance, and the Levels of Ethylene and Biotin Are at Least Partially Controlled by HvSAMS3 and Its Signaling Pathways

The upregulation of *SAMS3* might protect cells from stress by cytoskeletal reorganization and morphogenesis. In transgenic tobacco, the overexpression or downregulation of *SAMSs* results in a stunted plant phenotype with abnormal leaf coloration [69]. *HvSAMS3* silencing produced a decrease in plant height, dry weight, and K^+^ in XZ5 (Figure 3C). The results suggest that the plant growth and development of XZ5, as well as the subsequent increased shoot biomass and K^+^ absorption, are regulated by *HvSAMS3*. Additionally, the results suggest that reduced drought and salinity stress tolerance in BSMV:HvSAMS3-inoculated XZ5 plants is associated with reduced levels of Put, Spd, and Spm. These proteins play a significant role in plant growth and development by regulating the cell cycle, acting as cell signaling molecules that regulate plant tolerance to various abiotic stresses. Moreover, Spm, Spd, and Put can regulate the size of the potassium channel and the size of pores in the plasma membrane of guard cells, strongly modulating pore opening and closing and affecting stomatal regulation for plant growth and development (Figure 7). Thus, PAs can control water loss in plants to respond to the encountered stress [70]. Ethylene regulates germination, growth, development, and senescence in plants [71]. Our data showed that the BSMV-mediated silencing of HvSAMS3 plants reduced ethylene and biotin biosynthesis. The downregulation of the endogenous SAM pool has been shown to reduce ethylene production in transgenic tomato plants expressing a heterologous gene of SAM hydrolase that degrades SAM to form methylthioadenosine and homoserine [72]. Taken together, our findings demonstrate that the silencing of *HvSAMS3* affects Put, Spd, Spm, ethylene, and biotin during plant growth and development in XZ5.

We tested whether the application of exogenous ethylene and biotin affected SAMS3-mediated D+S stress tolerance. Here, the role of ethylene in plant growth and development has been widely documented under both normal and stress conditions [73]. Additional improvement in seedling growth with the co-application of ethylene and biotin in the BSMV:HvSAMS3-inoculated plants compared with the mock-inoculated barley plants indicates the coordinated interaction between ethylene and biotin in plant growth (Figure 5). SAMS catalyzes the only known reaction for the synthesis of SAM, which is a common precursor of ethylene and PA biosynthesis [74]. Although SAM is the precursor of ethylene, the key enzyme of ethylene synthesis is ACC (Figure 7) [67]. A lower magnitude of ethylene increment in BSMV:HvSAMS3-inoculated plants by ACC may be due to the limited availability of the downstream enzyme ACC oxidase compared with that in mock-inoculated barley plants (BSMV:γ). The stimulatory effect of ACC appears to be gene specific, because the treatment resulted in increased transcript accumulation of *HvSAMS3* but not in other members of the *HvSAMS* gene family. Although the precise role of ACC-induced SAMS expression may favor PA synthesis, exogenous ACC has been shown previously to upregulate PA accumulation in mustard [75]. Good et al. [72] reported that the reduction in the endogenous SAM pool by SAM hydrolase in transgenic tomato could result in decreased ethylene production. Thus, SAMS may play an important regulatory role in ethylene synthesis (Figure 7). Ethylene and biotin did not affect the normal growth of control seedlings; however, following D+S stress, plant growth was introverted more than in control seedlings (Figure 5), indicating that *SAMS3* is critical for D+S stress, suggesting that ethylene and biotin play a general role in the response to D+S stress and function downstream of SAMS3 in the SAM signaling pathway (Figure 7). Future elucidation of these specific transport systems at the biochemical and molecular levels will be helpful to elucidate the mechanisms of regulation of biotin synthesis and utilization in plants.

## 5. Conclusions

In summary, comparative proteomic analysis allowed us to identify the stress-responsive proteins associated with salinity and drought-stressed barley. Among the 21 proteins, HvSAMS3 was expressed differently between XZ5 and CM72 in response to combined drought and salinity. *HvSAMS3* was cloned, and its roles were characterized via BSMV-VIGS. BSMV-VIGS of *HvSAMS3* confirmed that this gene is involved in plant growth, PA synthesis, ethylene production, biotin production, and K^+^ uptake under both control and combined drought and salinity conditions. *HvSAMS3* is also regulated by exogenous ethylene and biotin under D+S stress. The reduced tolerance to the combined stress is associated with the inhibition of polyamine, polyamine oxidase, ethylene, biotin, and antioxidant enzyme activities. Our findings advance academic knowledge and may also result in potential economic benefits for molecular breeding of D+S-tolerant barley and other crops.

## Figures and Tables

**Table 1:** Proteins expression under D+S vs. drought stress: Proteins induced in XZ5 leaves (+) but downregulated (−)/unchanged in CM72, or unchanged in XZ5 but downregulated in CM72. (Uploaded: http://www.peptideatlas.org/PASS/PASS01597 Dataset tag: barley20200616).

Spot NO	Protein Name	Accession Number	MW (Da)	pI	MS	MP	AASC (%)	Fold Increase (+) or Decrease (−)			Function
								CM72	XZ16	XZ5	
C1	ATP synthase subunit beta, chloroplastic, [*Agrostis stolonifera*]	ATPB_AGRST	53,840	5.16	167	22	55	−2.0	+1.7	+2.9	Photosynthesis electron transfer
C2	Elongation factor Tu, chloroplastic-like [*Brachypodium distachyon*]	gi|357149925	50,638	5.88	681	16	34	+1.2	+1.5	+4.1	Protein synthesis
C3	Ribulose bisphosphate carboxylase small chain clone 512 (Fragment) [*Triticum aestivum*]	RBS3_WHEAT	13,275	5.84	187	7	53	+1.1	+2.2	+6.1	Photosynthesis Calvin cycle
C4	Predicted: 20 kDa chaperonin, chloroplastic-like (*B. distachyon*)	gi|357158586	26,043	6.19	74	6	48	+1.0	+1.7	+4.1	Defense/stress
C5	Ribulose bisphosphate carboxylase large chain [*Coleochaete orbicularis*]	RBL_COLOB	53,177	6.00	260	11	23	−1.0	+3.5	+1.9	Photosynthesis Calvin cycle
C6	Ribulose bisphosphate carboxylase large chain (Fragment) [*Rosa damascena*]	RBL_ROSDA	6090	4.81	173	5	86	−2.9	−2.6	+1.8	Photosynthesis Calvin cycle
C7	Putative chaperonnin 21 precursor [*Oryza sativa* Japonica]	gi|51090748	26,360	4.62	129	8	67	+1.6	+1.9	+3.0	Defense/stress
C8	Phosphoribulokinase, chloroplastic [*T. aestivum*]	KPPR_WHEAT	45,512	5.72	167	16	53	−2.5	+10^6^	+10^6^	Photosynthesis Calvin cycle
C9	Chlorophyll a-b binding protein 1, chloroplastic [*Sinapis alba*]	CB21_SINAL	28,328	5.17	95	7	25	−10^6^	+2.1	+10^6^	Photosynthesis electron transfer
C10	Ribulose bisphosphate carboxylase/oxygenase activase A, chloroplastic [*Hordeum vulgare*]	RCAA_HORVU	51,383	8.04	291	16	32	+1.4	+8.5	+9.8	Photosynthesis Calvin cycle
C11	Ribulose bisphosphate carboxylase large chain (Fragment) [*Galium lucidum*]	RBL_GALLU	50,674	6.58	112	10	29	−3.1	−3.5	+9.7	Photosynthesis Calvin cycle
C12	Cytosolic heat shock 70 protein; HSC70-3 [*Spinacia oleracea*]	gi|2642648	78,241	5.09	152	16	38	−1.8	−1.4	+3.3	Defense/stress
C13	Cytosolic heat shock protein 90 [*H. vulgare*]	gi|32765549	80,654	4.96	181	15	27	−1.6	+2.8	+4.5	Defense/stress

MS, Mascot score; MP, Matched peptide; AASC, Amino acid sequence coverage. Fold increase (+) or decrease (−) was calculated by drought vs. control. Protein spot ID refers to numbers in Figure 1C. Accession number of top database match from the NCBInr database. Fold increase and decrease were calculated as drought/control, and control/drought for up and downregulated proteins, respectively. All ratios shown are statistically significant (*p* < 0.05). +10^6^ and −10^6^ referred to the specific expressed and totally inhibited proteins, respectively.

**Table 2:** Proteins expression under D+S vs. salinity stress: Proteins induced in XZ5 leaves (+) but downregulated (−)/unchanged in CM72, or unchanged in XZ5 but downregulated in CM72. (Uploaded: http://www.peptideatlas.org/PASS/PASS01597 Dataset tag: barley20200616).

Spot NO	Protein Name	Accession Number	MW (Da)	pI	MS	MP	AASC (%)	Fold Increase (+) or Decrease (−)			Function
								CM72	XZ16	XZ5	
D1	Peroxiredoxin-2E-2, Chloroplastic [*O. sativa* sunsp. *Japonica*]	PR2E2_ORYSJ	23,279	6.15	154	8	19	−2.0	+2.6	+2.8	Redox homeostasis
D2	S-adenosylmethionine synthetase 3 [*H. vulgare*]	METK3_HORVU	43,138	5.51	129	15	44	−2.4	+1.7	+12.4	Primary metabolism
D3	Probable plastid-lipid-associated protein 3, chloroplastic [*O. sativa* subsp.]	PAP3_ORYSJ	40,073	4.42	75	2	3	−1.3	+2.2	+2.9	Primary metabolism
D4	Pyridoxal biosynthesis protein PDX1.1 [*Arabidopsis thaliana*]	PDX11_ARATH	33,126	5.75	189	12	26	−10^6^	−10^6^	+3.6	Intracellular traffic
D5	Light harvesting chlorophyll a/b-binding protein Lhcb1 [*H. vulgare*]	gi|297306780	38,271	6.26	96	9	39	−2.0	−3.6	+4.8	Photosynthesis electron transfer
D6	Chalcone-flavonone isomerase [*H. vulgare*]	gi|1705761	22,941	4.8	120	15	43	−1.3	−1.9	+7.9	Secondary metabolism

MS, Mascot score; MP, Matched peptide; AASC, Amino acid sequence coverage. Fold increase (+) or decrease (−) was calculated by drought vs. control. Protein spot ID refers to numbers in Figure 1D. Accession number of top database match from the NCBInr database. Fold increase and decrease were calculated as drought/control, and control/drought for up and downregulated proteins, respectively. All ratios shown are statistically significant (*p* < 0.05). +10^6^ and −10^6^ referred to the specific expressed and totally inhibited proteins, respective.

## Supplementary Materials

The following are available online at https://www.mdpi.com/2073-4409/9/6/1530/s1. All supplementary tables were uploaded on a publically available website: (Uploaded: http://www.peptideatlas.org/PASS/PASS01597 Dataset tag: barley20200616). Table S1: Candidate genes and corresponding primers used for qPCR experiments. Table S2: Primers used for VIGS and function verification. Table S3: Summary of identified SAMS proteins in different plant species. Table S4: Average leaf protein spots were resolved among the three genotypes (XZ5, XZ16, and cv. CM72) in response to drought, salinity stress alone, and their combination during vegetative stage at 4% soil moisture level. Table S5: Comparison of differentially drought and salinity responsive proteins in leaf of XZ5, XZ16, and CM72 genotypes in response to drought, salinity stress alone, and their combination during vegetative stage at a 4% soil moisture level. Table S6: Proteins whose expression was significantly induced (+) in XZ5 leaves but downregulated (−)/unchanged in CM72, or unchanged in XZ5 but downregulated in CM72 under drought stress (SMC 4%) vs. control. Table S7: Proteins whose expression was significantly induced (+) in XZ5 leaves but downregulated (−)/unchanged in CM72, or unchanged in XZ5 but downregulated in CM72 under salinity stress vs. control. Table S8: Proteins expression under D+S vs. drought stress and D+S vs. salinity stress: Proteins induced in XZ5 leaves (+) but downregulated (−)/unchanged in CM72 or unchanged in XZ5 but downregulated in CM72. Figure S1. Transmission electron micrograph of chloroplasts (A) and roots (B) of *cv.* CM72 (left panel) and Tibetan wild barley XZ16 (middle) and XZ5 (right) grown under control (a, b, c), drought (d, e, f), salinity alone (g, h, i), and combined stress D+S (j, k, l), respectively, during vegetative stage at the 4% soil moisture level. Bar = 0.2 μm. GL, granum lamellae; M, mitochondrion; Os, osmiophilic plastolobuli; SG; starch grain; SL, stroma lamellae; CW, cell wall; E, endoplasmic reticulum; N, nucleus; NL, nucleolus; P, plastid; PL, plasmalemma; V, vacuole. Figure S2. (A) Two-dimensional gel electrophoresis of protein extracted from barley leaf. (B) Venn diagram analysis of leaf protein spots differentially expressed among the three genotypes (XZ5 shown as yellow, XZ16 shown as cyan, and cv. CM72 shown as pink). Figure S3. ‘Spot view’ of the abundance of differentially expressed proteins (indicated with green circles) in leaves of three barley genotypes XZ5, XZ16 and CM72 under control, (A) drought stress (SMC 4%) and (B) salinity stress (200 mM NaCl). Figure S4. (A) Functional category distribution of the 60 identified differentially expressed proteins in this study. (B) Gene expression analyzed by qPCR. (C) The nucleotide and amino acid sequences of *HvSAMS3*. Figure S5. Multiple alignment of amino acid sequences of *AtSAMS1-AtSAMS4* and newly identified SAM in Barley (*HvSAMS1-HvSAMS4*) and representative monocot species. The conserved S-adenosylmethionine synthetase N-terminal domain and S-adenosylmethionine synthetase central domain are shown. Figure S6. Silencing of the phytoene desaturase (PDS) gene, (A) leaf phenotype and (B) relative expression in wild barley XZ5 using BSMV-VIGS. (C)Relative expression of *HvSAMS1, HvSAMS2* and *HvSAMS4* was measured by using real-time PCR. Error bars represent SD values (*n* = 3).
