# Correction: Ahmed et al. The Barley *S-Adenosylmethionine Synthetase 3* Gene *HvSAMS3* Positively Regulates the Tolerance to Combined Drought and Salinity Stress in Tibetan Wild Barley. *Cells* 2020, *9*, 1530

**DOI:** 10.3390/cells13191660

**Published:** 2024-10-08

**Authors:** Imrul Mosaddek Ahmed, Umme Aktari Nadira, Cheng-Wei Qiu, Fangbin Cao, Zhong-Hua Chen, Eva Vincze, Feibo Wu

**Affiliations:** 1 Department of Agronomy and Zhejiang Key Laboratory of Crop Germplasm, College of Agriculture and Biotechnology, Zijingang Campus, Zhejiang University, Hangzhou 310058, China; imrulbau@gmail.com (I.M.A.); kbdnadira@yahoo.com (U.A.N.); 3130100260@zju.edu.cn (C.-W.Q.); caofangbin@zju.edu.cn (F.C.); 2 Plant Physiology Division, Bangladesh Agricultural Research Institute, Gazipur 1701, Bangladesh; 3 Jiangsu Co-Innovation Center for Modern Production Technology of Grain Crops, Yangzhou University, Yangzhou 225009, China; 4 School of Science and Health, Hawkesbury Institute for the Environment, Western Sydney University, Penrith, NSW 2751, Australia; z.chen@uws.edu.au; 5 Department of Molecular Biology and Genetics, Aarhus University, Fosøgsvej 1, DK-4200 Slagelse, Denmark; eva.vincze@mbg.au.dk

## Error in Figure 2

In the original publication [1], there was a mistake in Figure 2. Spot views C1, C6, C7, C12, C13 and D6 were incorrect. This was an unintentional mistake. At the beginning, we serially numbered all protein spots and screened the protein spots for mass spectrometry analysis. However, when writing MS, in order to facilitate the readers’ comprehension of the results, we re-classified and numbered them according to traits such as tolerances to salt (S), drought (D) and D+S, which caused confusion and misalignment. Here, we once again express our deep apology. We thoroughly examined the current version in light of the original data and made the necessary revisions. As a result, we have changed spot views C1, C6, C7, C12, C13 and D6 in the revised paper. The corrected Figure 2 is shown below. 

**Figure 2:**
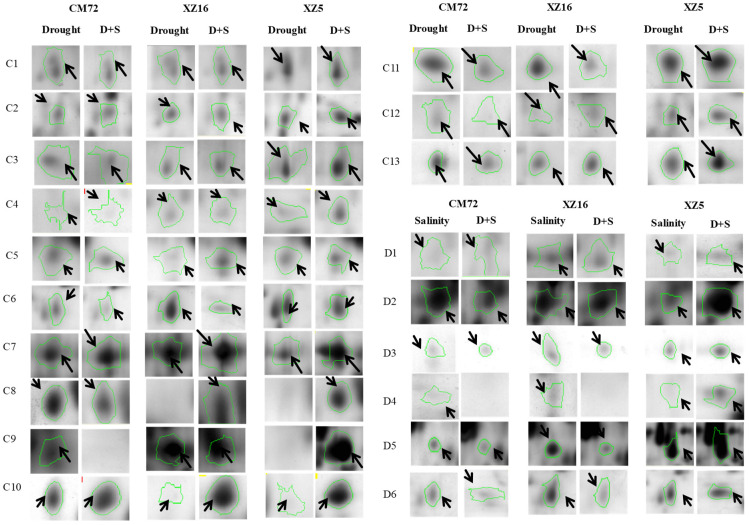
‘Spot view’ of the abundance of differentially expressed proteins (indicated with green circles) in the leaves of three barley genotypes XZ5, XZ16 and CM72 under drought stress, and salinity stress (200 mM NaCl) and D+S stress conditions. Protein spot IDs refer to numbers in Figure 1C,D and Tables 1 and 2.

## Error in Figure S2

In the original publication, there was a mistake in Figure S2. Some of the 2D gels in Figure 1 and Figure S2 were the same. For example, Figure 1A (XZ5 under drought) = Figure S2 (b) (XZ5 under drought) and Figure 1B (XZ5 under Salinity) = Figure S2 (c); for clarity and correctness, we have rearranged Figure S2. The corrected Figure S2 is shown below. 

**Figure:**
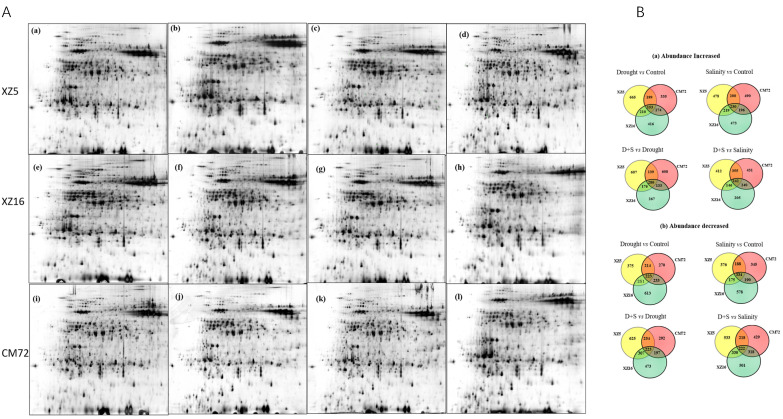


The authors state that the scientific conclusions are unaffected. This correction was approved by the Academic Editor. The original publication has also been updated.
